# Rapid testing with molecular syndromic panels of patients presenting at the emergency department with pneumonia at risk for multidrug-resistant pathogens

**DOI:** 10.1007/s10096-025-05376-2

**Published:** 2025-12-10

**Authors:** Lorenzo Pelagatti, Francesca Mangani, Elisa Muz, Lorenzo Corbetta, Sara Tomassetti, Alberto Farese, Alessandro Bartoloni, Francesca Caldi, Mattia Ronchetti, Tommaso Giani, Peiman Nazerian, Gian Maria Rossolini, Simone Vanni

**Affiliations:** 1 Emergency Department, Careggi University Hospital, Florence, Italy; 2 Department of Experimental and Clinical Medicine, University of Florence, Largo Brambilla 1, Florence, 50134 Italy; 3 Microbiology and Virology Unit, Careggi University Hospital, Florence, Italy; 4 Interventional Pulmonology Unit, Careggi University Hospital, Florence, Italy; 5 Infectious and Tropical Diseases Unit, Careggi University Hospital, Florence, Italy; 6 High Dependency Unit, Careggi University Hospital, Florence, Italy

**Keywords:** Molecular syndromic panels, Pneumonia, CAP-MDR, Antimicrobial therapy, Multi drug resistant pathogens

## Abstract

**Background:**

Pneumonia remains a major cause of morbidity and mortality worldwide. Rapid diagnostic testing with molecular syndromic panels (MSP) has proved useful for the management of antimicrobial treatment of hospital-acquired pneumonia (HAP). In this study, we evaluated the impact of early use of MSP testing in the Emergency Department (ED) for the management of antibiotic therapy in patients with moderate to severe CAP with risk factors for multi-drug-resistant pathogens (CAP-MDR).

**Patients and methods:**

Patients presenting at the ED with diagnosis of moderate to severe CAP-MDR underwent microbiological analysis of lower respiratory tract specimens by culture and MSP testing (bioMérieux, FilmArray^®^ Pneumonia Plus Panel). The primary outcome was the percentage of cases in which MSP testing modified the empiric antimicrobial therapy started according to local protocols. Among the secondary outcomes we included the time elapsed from ED admission to antibiotic change or confirmation upon receipt of the MSP results.

**Results:**

Between June 2024 and May 2025, 93 patients were enrolled (age, 72.9 ± 13.9 years; 62.4% males). MSP testing identified one or more pathogens in 91.4% of cases. Modification of empiric antibiotic therapy, started according to local protocols, occurred in 65.6% of patients (escalation in 44.1% and de-escalation in 21.5% of cases) after 11.4 ± 6.3 h (IQR 12.0) since ED presentation.

**Conclusions:**

The early use of MSP on lower respiratory samples collected in the ED from patients with diagnosis of moderate to severe CAP-MDR could allow a rapid and targeted modification of the empiric antibiotic therapy in these patients, with potential advantages on antimicrobial stewardship and patient management.

**Supplementary Information:**

The online version contains supplementary material available at 10.1007/s10096-025-05376-2.

## Introduction

Pneumonia remains a leading cause of morbidity and mortality worldwide, with more than 3.1 million deaths annually. It ranks as the eight most common cause of death, being also a primary trigger for sepsis [1, 2]. Hospitalized cases are associated with high mortality rates [3]. Among the most challenging forms to treat are hospital-acquired pneumonia (HAP) and community-acquired pneumonia (CAP) with risk factors for multidrug-resistant pathogens (CAP-MDR), previously defined as healthcare-associated pneumonia (HCAP), which are often caused by difficult-to-treat MDR pathogens [4, 5]. CAP-MDR occurs in non-hospitalized patients with risk factors for MDR pathogens including hospitalization for at least two days within the previous 90 days, residence in nursing homes or long-term care facilities, home infusion therapy including antibiotic therapy, chronic dialysis, home wound care, and contact with individuals colonized by MDR organisms [1, 6]. The primary etiologic agents of CAP-MDR are MDR pathogens including Gram-negative bacilli (e.g. Enterobacterales producing extended-spectrum β-lactamases or carbapenemases, *Pseudomonas aeruginosa* with difficult-to-treat resistance (DTR), and carbapenem-resistant *Acinetobacter baumannii*) and methicillin-resistant *Staphylococcus aureus*, while viral and fungal pathogens are rarely involved in immunocompetent patients [7, 8].

CAP is a significant cause of emergency department (ED) visits and hospital admissions, and in CAP-MDR the empiric treatment is often inappropriate [9, 10]. In this regard, the IDSA 2019 guidelines for CAP recommend against initiating empirical therapy based on the old definition of HCAP, which is often excessive and inadequate and was not shown to provide clinical benefit, underscoring the importance of microbiological diagnosis [11–14]. However, conventional diagnosis by culture typically requires at least two days to provide results.

Recently, diagnostic systems for lower respiratory tract infections based on molecular syndromic panels (MSP) have become available, which allow the rapid detection of multiple pathogens and of clinically relevant resistance mechanisms with high sensitivity, and can usefully complement conventional culture-based diagnostics [1]. Indeed, previous studies with MSP, predominantly conducted in the ICU setting [15–17], have shown a clear impact on antimicrobial stewardship for treatment of HAP [18–27], although they have not shown a significant effect on clinical outcomes such as mortality, length of hospital stay, or readmission rates [25–27]. Conversely, data regarding the use of MSP testing for patients with CAP-MDR in the ED setting are currently lacking and the impact of implementation of MSP testing in this setting warrants further investigation.

This study aimed to evaluate the impact of early use of an MSP (bioMérieux, FilmArray^®^ Pneumonia Plus Panel) for the management of antibiotic therapy in patients with moderate to severe CAP-MDR presenting to the ED.

## Patients and methods

### Study design

The study was designed as a prospective, single-centre, single-arm cohort, non-profit, observational study that enrolled consecutive adult patients presenting to the ED, diagnosed with moderate to severe CAP-MDR, and undergoing diagnostic testing by both rapid MSP and conventional standard-of-care approach. The study design did not foresee randomization nor included a control group; all enrolled patients underwent both MSP testing and conventional respiratory cultures.

The hospital where the study was carried out is a large tertiary-care teaching hospital located in Florence, Italy (an area of high endemicity of MDR pathogens [28]), that functions as a referral hospital for approximately one million inhabitants and as a local hospital for about 650,000 people.

The study took place in the ED and the High Dependency Unit (HDU), in collaboration with the hospital’s Units of Microbiology and Virology, Interventional Pulmonology, and Infectious Diseases. The ED and the HDU were responsible for patient enrolment, clinical evaluation, respiratory sample collection, and data collection. The Microbiology and Virology unit processed samples for microbiological diagnosis. The Infectious Diseases Unit assessed the appropriateness of antimicrobial therapy based on results of MSP testing and advised about the need for escalation or de-escalation of empiric therapy started according to local protocols (eFigure 1e). The Interventional Pulmonology Unit assisted in collecting respiratory samples through BAL, BAS or ETA procedures, in collaboration with the ED and HDU.

**Fig. 1:**
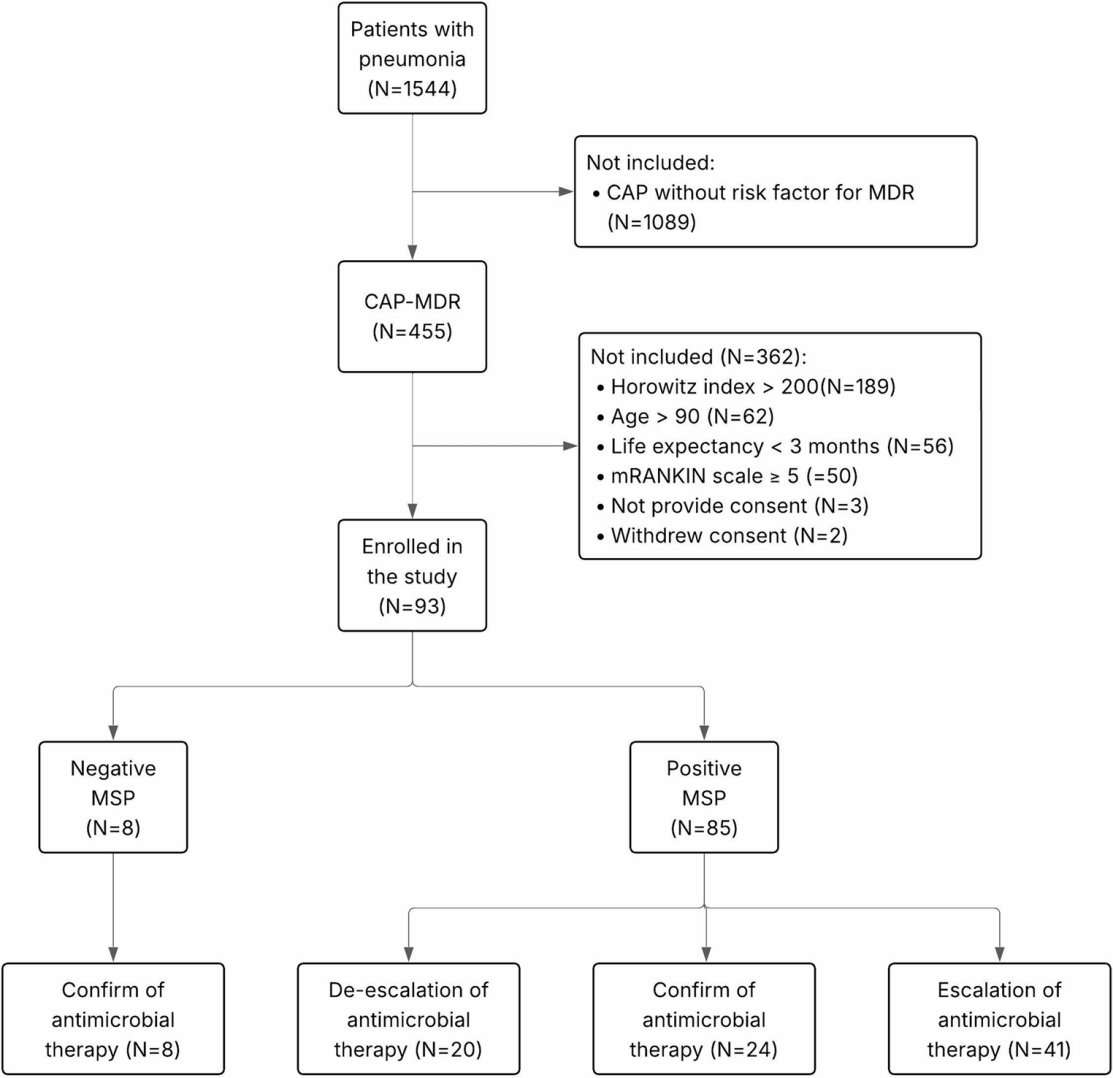
Study flow chart showing the patients workup and the steps from the initial assessment of eligibility to the final analysis of the 93 patients included in the study

All patients were followed up for 30 days. Follow-up data were obtained from electronic medical records or via telephone contact, when necessary.

### Inclusion criteria

Patients aged ≥ 18 years presenting to the ED with a clinical and radiological diagnosis of pneumonia and undergoing respiratory sampling (BAL, BAS, or ETA) were considered eligible if they had a pre-morbid modified Rankin Score (mRS) < 5 [29]. Pneumonia was defined according to IDSA guidelines as the presence of a new pulmonary infiltrate on chest radiograph and at least two of the following: fever >38 °C, leucocytosis or leukopenia, and purulent secretions [1]. Eligible patients were enrolled when classified as having moderate to severe CAP-MDR, defined in the presence of at least one of the following risk factors: (a) hospitalization in an acute-care hospital for ≥ 2 days within 90 days prior to symptom onset; (b) residence in a nursing home or long-term care facility; (c) recent (within 30 days) intravenous antibiotic therapy, cancer chemotherapy, or wound care; (d) attendance at a hospital or haemodialysis clinic within the last 30 days; (e) immunosuppression. In addition one of the following severity criteria had to be present: (i) requirement for high-flow oxygen therapy (e.g., VM35, FiO₂ >0.35, reservoir mask, high-flow nasal cannula - HFNC - or non-invasive ventilation - NIV); (ii) endotracheal intubation (IOT); (iii) SOFA score ≥ 2 (respiratory component) or Pneumonia Severity Index (PSI) >85 (e.g., age >75 + respiratory impairment); (iv) Horowitz index (PaO₂/FiO₂) < 200.

Exclusion criteria included lack of informed consent, age < 18 or > 90 years, pregnancy, life expectancy < 3 months, ED admission since > 24 h, and modified Rankin Score ≥ 5.

### Study outcomes

The primary outcome was to evaluate the impact of early use of MSP on empirical antimicrobial therapy for CAP-MDR in terms of revision of the empiric regimen selected according to the hospital protocols (eFigure 1e). This was evaluated by measuring the proportion of patients whose empiric antibiotic therapy was modified—either through escalation or de-escalation—within 24 h of ED admission, based on the MSP results. *Escalation* was defined as broadening the antibiotic spectrum, either by adding another agent or by switching to a broader spectrum drug presumably active against the identified pathogen. *De-escalation* was defined as narrowing the antibiotic spectrum by discontinuing unnecessary agents or switching to a narrower spectrum drug.

The secondary outcomes included: time (in hours) from ED admission to respiratory sample collection and to empiric treatment re-evaluation leading to modification (escalation/de-escalation) or confirmation; rate of etiological diagnosis (within 7 days) using all available methods (MSP and SOC diagnostics); length of hospital stay (in days); need for escalation of supportive care, defined as need for non-invasive or invasive ventilation, transfer to a higher level of care (medical ward → sub-intensive → intensive care) or in-hospital mortality from any cause (“complicated course”); duration of antibiotic therapy (in days); de-escalation or escalation of antimicrobial therapy within 7 days of enrolment; in-hospital mortality due to pneumonia and all causes; 14 and 30-day mortality (all causes and pneumonia-specific), and rehospitalization.

Safety outcomes included complications related to lower respiratory tract sample collection and time (in hours) from sample collection to administration of pathogen-targeted therapy, measured using electronic medical records.

### Patients diagnostic workup

Patients enrolled in the study underwent a comprehensive diagnostic workup, including arterial blood gas analysis, laboratory testing, imaging (chest X-ray, lung ultrasound, and computed tomography) and microbiological analysis of lower respiratory tract specimens (BAL, BAS or ETA) by conventional culture (supplemental material) and by MSP testing using a commercial system (FilmArray^®^ Pneumonia Plus Panel, bioMérieux) according to the manufacturer’s instructions [30]. The MSP detects 18 bacterial pathogens (including 11 Gram-negative, 4 Gram-positive, and 3 atypical organisms), 7 antibiotic resistance genes, and 9 respiratory viruses (Supplemental Table S1) with a turnaround time of approximately 75 min. Results for typical Gram-positive and Gram-negative bacterial pathogens are reported semiquantitatively, as genomic copies/ml (REF). Additional microbiological investigations, including blood cultures, urinary antigen testing for *Streptococcus pneumoniae* and *Legionella pneumophila*, serum β-D-glucan testing, *Aspergillus* galactomannan assay on BAL fluid, and molecular tests for the detection of additional viral pathogens (e.g. SARS-CoV-2, herpesviruses) and fungal pathogens (e.g. *Aspergillus spp.* and *Pneumocystis jirovecii*) not targeted by the MSP were ordered when deemed appropriate (Supplementary material).

In all patients, respiratory specimens were collected and processed using both the MSP and the reference standard methods (Supplementary material).

Antimicrobial therapy was initiated empirically according to local protocols (eFigure 1e) and subsequently revised based on MSP results or, in selected cases, directly started as targeted therapy following MSP results. When MSP detected pathogens that did not require Gram-positive coverage (e.g. *K. pneumoniae* without concomitant Gram-positive organisms), agents targeting Gram-positive pathogens (e.g., linezolid or vancomycin) were discontinued. Therapeutic decisions were made on a case-by-case basis, according to pathogen-specific coverage needs and after consultation with an infectious disease specialist. In accordance with antimicrobial stewardship principles, unnecessary antibiotics were systematically withdrawn to optimize targeted therapy.

### Sample size and statistical plan

Based on data from previous studies conducted in intensive care settings, we anticipated that the antibiotic therapy might be modified in at least 40% of cases. Assuming a study power of 80% and a margin of error of ± 10%, the estimated sample size required for the study was calculated to be 93 patients.

### Statistical analyses

Quantitative and qualitative variables were described within subgroups (with or without modification of antibiotic therapy) using median and interquartile range, or—when appropriate—mean and standard deviation, and absolute and relative frequencies, respectively. The distribution of continuous variables was preliminarily assessed using Q-Q plots and the Shapiro-Wilk test.

Differences between two groups for quantitative variables were evaluated using the Student’s *t*-test for parametric data or the Mann-Whitney test for non-parametric data. In the case of comparisons involving more than two groups, the ANOVA test (parametric) or the Kruskal-Wallis test (non-parametric) was applied, with Bonferroni correction for multiple comparisons. For qualitative variables, Fisher’s exact test was used. A two-tailed p-value < 0.05 was considered statistically significant. All analyses were performed using the SPSS statistical software (SPSS 2019).

### Ethical clearance

The study protocol received approval from the local ethics committee (CEAVC 26198_oss), and all participants provided informed consent in accordance with the Declaration of Helsinki. Patients were not involved in the design of this study. The study has been registered at ClinicalTrials.gov (NCT06506617).

## Results

Between June 1 st, 2024, and May 1 st, 2025, a total of 93 consecutive patients with CAP-MDR responding to the enrolment criteria were included in the study (Fig. 1). The mean age of the study population was 72.9 ± 13.9 years. Female patients accounted for 37.6% of the cohort (Table 1).

**Table 1:** Patients characteristics, comorbidities, vital signs, arterial blood gas analysis, laboratory tests. COPD: chronic obstructive pulmonary disease; CKD: chronic kidney disease; HR: heart Rate; SBP: systolic blood Pressure; DBP: diastolic blood Pressure; SpO₂: oxygen Saturation; FiO₂: fraction of inspired oxygen; GCS: Glasgow coma Scale; BT°C: body temperature (°C); WBC: white blood cell Count; hb: Hemoglobin; hct: Hematocrit; CRP: C-reactive Protein; PCT: Procalcitonin; BUN: blood Urea nitrogen

PATIENTS CARACTERISTICS		POPULATION (*n* = 93)		CONFIRMATION (*n* = 32)		ESCALATION (*n* = 41)		DE-ESCALATION (*n* = 20)	*P* (escalation vs. others)
Age		72.9 ± 13.9		72.5 ± 15.0		74.6 ± 12.4		70.1 ± 15.5	0.49
Female sex		35/93 (37.6%)		13/32 (40.6%)		14/41 (34.1%)		8/20 (40%)	0.83
Comorbidities									
COPD		32/93 (34.4%)		8/32 (25.0%)		18/41 (43.9%)		6/20 (30.0%)	0.22
Asthma		4/93 (4.6%)		3/32 (9.4%)		1/41 (2.4%)		0/20 (0.0%)	0.18
Diabetes mellitus		21/93 (22.6%)		7/32 (21.9%)		10/41 (24.4%)		4/20 (20.0%)	0.92
Bronchiectasis		2/93 (2.2%)		1/32 (3.1%)		1/41 (2.4%)		0/20 (0.0%)	0.74
Stroke		13/93 (14.0%)		1/32 (3.1%)		11/41 (26.8%)		1/20 (5.0%)	***0.01***
CKD		20/93 (21.5%)		8/32 (25.0%)		9/41 (21.9%)		3/20 (15.0%)	0.69
Immunosuppresion		21/93 (22.6%)		11/32 (34.4%)		3/41 (7.3%)		7/20 (35.0%)	***0.01***
Vitals									
HR	94.5 ± 22.4		98.7 ± 24.3		93.1 ± 20.8		90.7 ± 22.6		0.41
SBP	120.6 ± 25.0		122.3 ± 24.4		116.2 ± 26.6		125.5 ± 22.4		0.34
DBP	67.9 ± 13.0		68.8 ± 10.7		65.3 ± 15.4		71.9 ± 10.3		0.15
SpO2	92.6 ± 6.4		92.3 ± 5.2		92.10 ± 7.6		94.2 ± 5.2		0.44
GCS	14.2 ± 2.3		14.9 ± 0.1		13.7 ± 2.9		14.0 ± 2.4		**0.05**
BT°C	37.3 ± 1.0		37.6 ± 1.01		37.0 ± 0.9		37.7 ± 1.1		**0.02**
Arterial blood gas analysis									
pH	7.43 ± 0.74		7.45 ± 0.05		7.41 ± 0.10		7.43 ± 0.05		0.08
pO2 mmHg	74.2 ± 55.0		67.3 ± 30.9		77.1 ± 69.4		79.3 ± 53.1		0.68
pCO2 mmHg	38.8 ± 14.5		34.6 ± 6.3		42.3 ± 19.0		38.3 ± 11.9		0.08
Lactate mg/dL	1.9 ± 1.7		1.9 ± 1.6		2.1 ± 1.9		1.5 ± 1.1		0.49
FiO2	35.9 ± 21.6		31.4 ± 16.1		40.9 ± 24.7		31.8 ± 21.2		0.13
Laboratory test									
WBC*10^9	12.5 ± 7.90		11.6 ± 8.0		13.9 ± 8.3		11.0 ± 6.8		0.30
Hb g/dL	11.7 ± 2.5		12.3 ± 2.2		11.3 ± 2.2		11.7 ± 2.3		0.19
Hct %	35.9 ± 6.5		37.6 ± 6.3		34.9 ± 6.5		35.6 ± 6.8		0.21
PCR mg/L	112.0 ± 97.1		101.7 ± 95.8		110.7 ± 91.9		131.2 ± 110.8		0.57
PCT ng/mL	7.9 ± 42.9		2.1 ± 5.2		12.4 ± 62.8		7.8 ± 21.8		0.60
Creatinine mg/dL	1.2 ± 0.8		1.2 ± 0.7		1.2 ± 0.9		1.3 ± 1.0		0.84
BUN g/L	1.0 ± 0.4		0.9 ± 0.4		1.1 ± 0.6		1.0 ± 0.3		0.59
Bilirubin mg/dL	0.6 ± 0.4		0.7 ± 0.4		0.6 ± 0.4		0.7 ± 0.4		0.59
Sodium mEq/L	138.7 ± 6.0		137.1 ± 6.3		140.3 ± 5.6		137.8 ± 6.1		0.06

All enrolled patients underwent lower respiratory sample collection for microbiological testing by conventional culture and by MSP. Respiratory specimens consisted of BAL in 93.6% of cases (87/93), all obtained by emergency physicians, BAS in 3.2% (3/93), and ETA in 3.2% (3/93). All three ETAs were collected from patients with tracheostomy cannulas. Sampling was performed in the Emergency Room in 80.6% of patients (75/93) and in the HDU in the remaining cases.

The respiratory samples for MSP and cultural testing were collected after a mean time of 8.1 ± 6.3 h (IQR 12.0) since presentation to the ED. Overall, MSP testing detected ≥ 1 pathogen in most cases (*N* = 85, 91.4%). A polymicrobism was detected in 55 cases (59.1%). Resistance genes were identified in 35 cases (37.6%) (Table 3). Results of conventional culture on the same specimens were returned after a mean time of 92.4 ± 40.2 h (IQR 49.3) since presentation to the ED. Additional microbiological investigations (e.g. blood cultures, urinary antigen testing for *S. pneumoniae* and *L. pneumophila*, and tests for the detection of additional viral and fungal pathogens not targeted by the MSP were ordered as reported in Table S2 (Supplementary material).

Following admission to the ED and confirmation of diagnosis of CAP-MDR, all enrolled patients received empiric antimicrobial treatment according to the hospital protocols. When results of MSP testing became available, the antimicrobial treatment originally prescribed was changed in 61 cases (65.6%, CI 95% 55.0–75.1.0.1) and confirmed in 32 cases (34.4%, CI 95% CI 95% 24.9–44.9). The changes consisted in escalation in 41 cases (44.1%, CI 95% 33.8–54.8) and de-escalation in 20 cases (21.5%, CI 95% 13.7–31.2). Escalation was in 29 cases (31.2%) to cover bacterial pathogens, in 10 cases (10.7%) to cover viral pathogens, and in two cases (2.1%) for combined coverage of bacterial and viral infections. Therapy was de-escalated in 19 cases (20.4%) to reduce unnecessary broad-spectrum coverage of bacterial pathogens, and in one case (1.1%) to cover an atypical pathogen (*Legionella spp*). Antibiotic therapy was confirmed in 24 cases since the original empiric regimen matched the identified pathogens, and in 8 cases since no pathogens were detected by the MSP. In the latter cases empirical therapy was continued as per local protocols until results of the culture testing and subsequently de-escalated in three cases and confirmed in the remaining five cases.

The mean time from ED admission to antimicrobial therapy revision upon receipt of the MSP testing results was 11.4 ± 6.3 h (IQR 12.0) with a mean time-to-response of 3.2 ± 2.3 h (IQR 0.6).

Comorbidities were analyzed across the subgroups in which MSP testing led to escalation, de-escalation, or confirmation of empirical antibiotic therapy, as shown in Table 1. Furthermore, Table 1 shows the values ​​of vital signs, arterial blood gas analysis values ​​and laboratory tests. Among comorbidities only the presence of stroke was significantly more frequent in the escalation group. This finding well matched with the lower GCS observed in the escalation group, in comparison to the others. Unexpectedly, immunosuppression was less frequent in the escalation group. All risk factors for MDR pathogens showing a significant difference among groups were more prevalent in the escalation group, except for recent chemotherapy (Table 2).

**Table 2:** Main Risk Factors for MDR Pathogens. NGT: nasogastric tube; NET: nasoenteric tube; PEG: Percutaneous endoscopic gastrostomy; PPI: Proton pump inhibitor; MRSA: Methicillin-resistant Staphylococcus aureus; NIV: Non-invasive ventilation; OTI: orotracheal intubation. §: Hospital visitor for medical reasons, outpatient visits, and day services

RISK FACTORS FOR MDR GERMS	POPULATION (*n* = 93)	CONFIRMATION (*n* = 32)	ESCALATION (*n* = 41)	DE-ESCALATION (*n* = 20)	*P* (escalation vs. others)
Hospitalized for 2 or more days within the past 90 days ^*,**^	60/93 (64.5%)	22/32 (68.7%)	31/41 (75.6%)	7/20 (35.0%)	***0.01***
^*^ Length of hospital stay (days)	17.1 ± 19.2	11.5 ± 7.1	22.3 ± 24.7	11.3 ± 4.1	***0.04***
^**^ Time since discharge (days)	30.7 ± 24.2	38.7 ± 27.7	27.8 ± 23.4	25.6 ± 22.6	0.24
Nursing home resident	33/93 (35.5%)	6/32 (18.7%)	23/41 (56.1%)	4/20 (20.0%)	***< 0.001***
Intravenous therapy within 30 days	50/93 (53.8%)	12/32 (37.5%)	30/41 (73.2%)	8/20 (40.0%)	***0.01***
Chemotherapy within 30 days	17/93 (18.3%)	8/32 (25.0%)	3/41 (7.3%)	6/20 (30.0%)	***0.04***
Wound care	15/93 (16.1%)	5/32 (15.6%)	9/41 (21.9%)	1/20 (5.0%)	0.24
Frequent hospital visitor ^§^	49/93 (52.7%)	20/32 (62.5%)	16/41 (39.0%)	13/20 (65.0%)	0.06
Antibiotic therapy within 30 days	44/93 (47.3%)	9/32 (28.1%)	29/41 (70.7%)	6/20 (30.0%)	***< 0.001***
Antibiotic therapy within 60 days	54/93 (58.1%)	17/32 (53.1%)	30/41 (73.2%)	7/20 (35.0%)	***0.01***
Tracheostomy	13/93 (14.0%)	0/32 (0.0%)	10/41 (24.4%)	3/20 (15.0%)	***0.01***
NGT/NET/PEG	13/93 (14.0%)	1/32 (3.1%)	11/41 (26.8%)	1/20 (5.0%)	***0.01***
Drug-resistant pneumonia within 1 year	14/93 (15.1%)	3/32 (9.4%)	9/41 (21.9%)	2/20 (10.0%)	0.25
Hospitalization within 60 days	51/93 (54.8%)	19/32 (59.4%)	27/41 (65.8%)	5/20 (25.0%)	***0.01***
PPI within 14 days	68/93 (71.1%)	21/32 (65.6%)	34/41 (82.9%)	13/20 (65.0%)	0.17
MRSA colonization within 1 year	2/93 (2.2%)	1/32 (3.1%)	1/41 (2.4%)	0/20 (0.0%)	0.74
NIV/OTI < 90 gg	10/93 (10.8%)	1/32 (3.1%)	8/41 (19.5%)	1/20 (5.0%)	***0.05***
Karnofsky performance status < 70	43/93 (46.2%)	10/32 (31.2%)	26/41 (63.4%)	7/20 (35.0%)	***0.01***

### Pathogen identification and comparison of MSP testing with culture results

Pathogen identification by any method (including MSP testing, conventional cultures, and additional tests) within seven days of enrolment was overall achieved in 89/93 patients (95.7%).

**Fig. 2:**
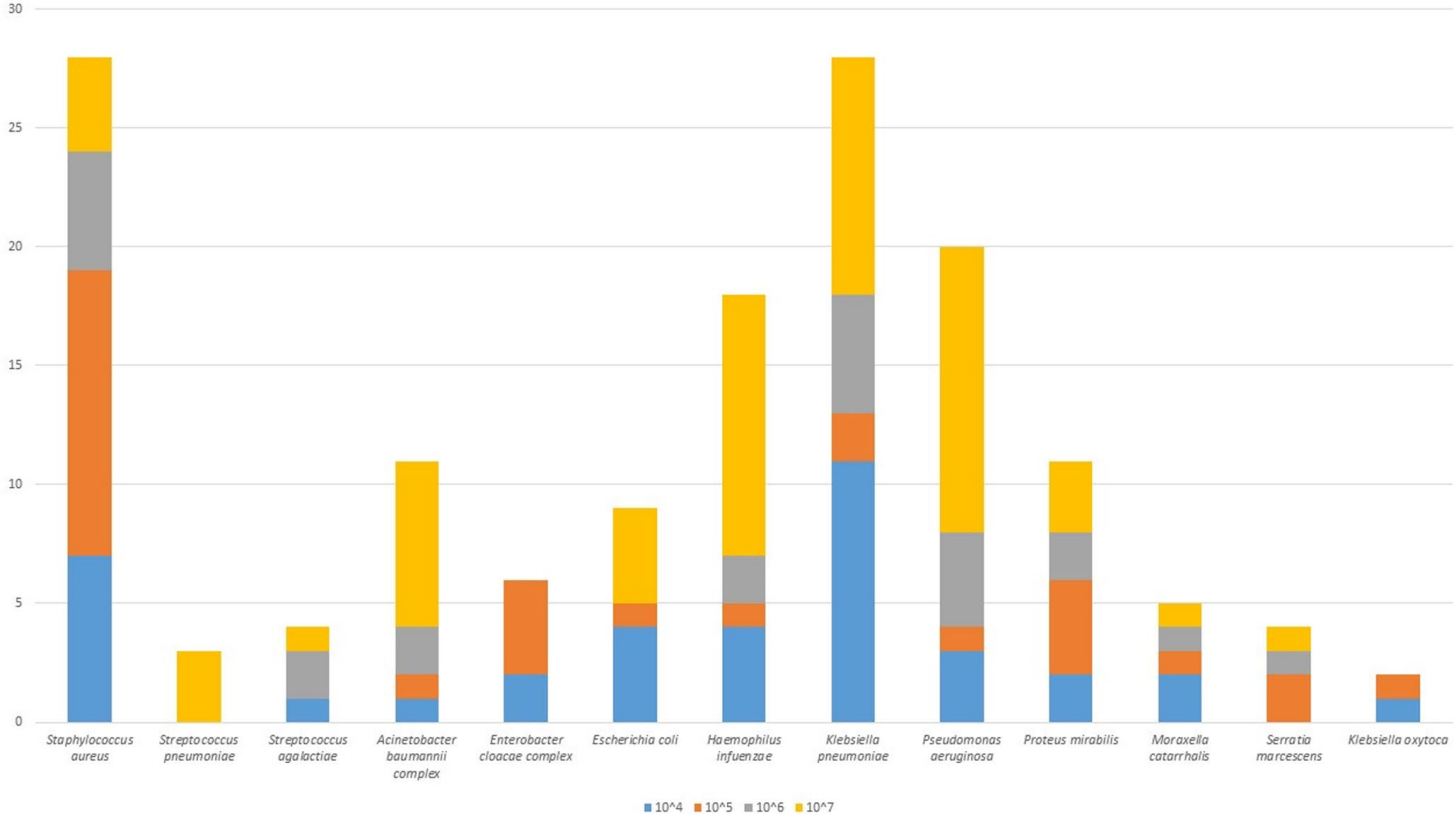
Bacterial targets detected by MSP testing according to semiquantitation of gene copy numbers. (In 1 case gene copy numbers of *S.aureus*,* A.baumannii complex*,* H.influenzae*,* S.pneumoniae* was not available and was not reported in fig S1)

MSP testing detected at least one bacterial pathogen in 85 cases (91.4%). In these, polymicrobism was detected in 55 cases (59.1%). Of the 154 bacterial pathogens detected by MSP, the five most prevalent were *Staphylococcus aureus* (*n* = 29, 18.8%), *Klebsiella pneumoniae* (*n* = 28, 18%), *Pseudomonas aeruginosa* (*n* = 20, 13.0%), *Haemophilus influenzae* (*n* = 19, 12.3%), and *Acinetobacter baumannii complex* (n *=* 12, 7.8%) (Fig. 2).

Resistance determinants in combination with usually associated bacterial pathogens were detected in 32 cases (34.4.6%), while in 3 cases a resistance determinant was detected, but in absence of a usually associated pathogen. In particular, a methicillin-resistance determinant along with *S. aureus* was detected in 11 cases (31.4%), a KPC carbapenemase determinant (either alone or in combination with a CTX-M ESBL determinant) along with *Enterobacterales* (mostly *Klebsiella pneumoniae*) was detected in 14 cases (40%), a CTX-M ESBL determinant along with Enterobacterales was detected in 11 cases (29.7%), and, a VIM-type metallo-β-lactamase determinant in the presence of *Enterobacterales* or *P. aeruginosa* was detected in two cases (5.7%), while the presence of OXA-48 and NDM determinants (either alone or in combination with other determinants) was not detected (Table 3).

**Table 3:** Resistance genes detected by MSP testing in the studied population. * In 3 cases (1 *P. aeruginosa* and 2 *A. baumanii *) a resistance determinant not correlated with a bacterial pathogen putatively associated with that determinant was reported by the system

Resistance determinants	(*N* 41)	Putatively associated bacterial pathogens (*N* 38)
MecA-MreJ	11	*S. aureus* (11)
CTX-M	13	*Enterobacterales* (11^*^)
CTX-M + KPC	14	*Enterobacterales* (13*)
KPC	1	*Enterobacterales* (1)
VIM	2	*Enterobacterales* (1) + *P. aeruginosa* (1)

Among atypical bacteria, only one *Legionella pneumophila* was identified, while *Chlamydia pneumoniae* and *Mycoplasma pneumoniae* were not detected.

Regarding viral pathogens, 38 viral targets were identified by MSP testing in 31 cases. The commonest virus detected was influenza A (*n* = 11, 29%), followed by rhinovirus/enterovirus (*n* = 10, 26.3%), non-SARS coronavirus (*n* = 9, 23.6%), parainfluenza virus (*n* = 6, 15.7%) and metapneumovirus (*n* = 2, 5.2%). Influenza B, respiratory syncytial virus, adenovirus and MERS-CoV were not found.

**Fig. 3:**
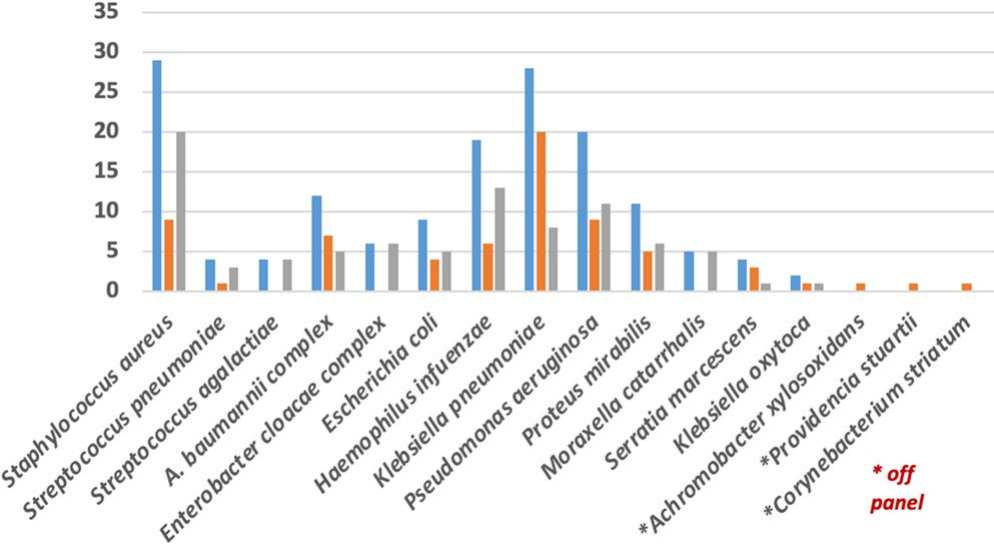
Detection of bacterial pathogens by MSP testing and by culture in the LRT samples from the 93 CAP-MDR patients. Blue: pathogen detected by MSP testing and/or culture; orange: pathogen detected only by culture; grey: pathogen detected only by MSP testing. Pathogens labelled by an asterisk are not included as targets in the MSP

Conventional cultures from the same respiratory samples used for MSP testing were positive for bacterial pathogens in 60 cases (64.5%), with polymicrobism detected in 20 cases (21.5%). At least one MDR organism was detected in 35 cases (37.6%). As expected, the detection rate of bacterial pathogens by MSP was overall higher than that by culture, although in some cases pathogens not targeted by the MSP were only detected by culture, including *Achromonacter xylosoxidans*, *Providencia stuartii* and *Corynebacterium striatum* (one case each, Fig. 3). In 44 cases the respiratory samples for microbiological investigation were collected before administration of antibiotic therapy, while in 49 cases after initiation of empirical antibiotic therapy: the rates of cultural positivity for bacterial pathogens were significantly higher in the former group (68.2% vs. 48.9%; *p* < 0.01) (Fig. 3).

Concerning additional antigenic testing, SARS-CoV-2 was also searched in all cases, with a positivity rate of 5.4% (5/93). Urinary antigens for *Streptococcus pneumoniae* were searched in 71/93 cases (76.3%) and were positive in 9 cases (9.6%). Of these, two were confirmed by MSP and one also by culture. Urinary antigens for *Legionella pneumophila* were searched in 70/93 cases (75%) and were positive in 2 cases (2%). Of these, one was confirmed by MSP.

Blood cultures were ordered in 58 cases and positive in 11 of them (18.7%), for various bacterial or fungal pathogens. In 4 of these cases (2 *S. aureus*, 1 *A. baumannii* complex, 1 *E. coli*), the pathogens isolated from blood were consistent with those detected in the lower respiratory tract (supplementary material – Table S3).

Galactomannan antigen and *Aspergillus* DNA were searched in 28 and 30 cases, respectively, with a positivity rate of 19.3% (18/93) and 9.6% (9/93), respectively. Finally, *Pneumocystis* spp. DNA was searched in 27 cases, with a positivity rate of 9.6% (9/27). These results lead to an escalation of antimicrobial therapy by adding an antifungal drug.

### In hospital course and follow-up

The mean duration of antibiotic therapy was 7.7 ± 2.9 days and the mean hospital stay was 13.5 ± 10.1 days. A total of 26 out of 93 patients (28%) required escalation of supportive therapy (need for invasive or non-invasive ventilation, transfer to a higher-intensity care unit, in-hospital death from all causes, or complicated clinical course): specifically, (a) new need for invasive or non-invasive ventilation in 4/93 (4.3%), (b) transfer to a higher-intensity care unit in 2/93 (2.1%), (c) new need for vasopressor support in 4/93 (4.3%), and (d) in-hospital death from all causes (complicated course) in 24.7% (23/93). Further de-escalation of antibiotic therapy within the first 7 days from inclusion occurred in 2 patients (2.2%), while escalation was necessary in 10 out of 93 patients (10.8%).

Overall, the observed 14-day mortality was 12.9% (*N* = 12/93) while the 30-day mortality in the population was 25.8% (*N* = 24/93). In-hospital mortality due to pneumonia and all causes was 24.7% (23/93), while 30-day mortality due to other causes was 1.1% (1/93). The 30-day readmission rate was 5.4% (5/93).

### Safety outcomes

No complications related to the collection of tracheobronchial aspirates and/or BAL were observed. The mean time from the collection of respiratory samples to the administration of pathogen-targeted antibiotic therapy was 3.2 ± 2.3 h (IQR 0.6).

## Discussion

This study demonstrated that in patients with moderate to severe CAP-MDR presenting to ED, early testing of lower respiratory tract specimens using an MSP is feasible and safe and can prompt rapid revision of empirical antimicrobial therapy as escalation or de-escalation in more than 60% of cases. With regard to the detection rate, a good concordance between MSP and conventional cultures was observed, although the MSP showed a tendency toward overdiagnosis compared with culture results (Fig. 3). To our best knowledge, this is the first study to evaluate the early use of MSP in the ED with patients with CAP and risk factors for MDR pathogens. In our study only five (5%) patients refused to participate. In the other cases, a lower respiratory sample collection was feasible, timely (in about three hours) and safe (no major complications occurred).

Currently, only one published study has assessed MSP testing in the ED among pneumonia patients. Markussen et al. [31] evaluated its performance in CAP cases in Norway, comparing it with conventional methods plus SARS-CoV-2 PCR. Their main aim was not to examine antibiotic management, but to assess diagnostic yield. MSP identified at least one clinically relevant pathogen in 81% of CAP cases, significantly increasing etiologic diagnosis compared with conventional methods. Similarly, in our cohort, MSP detected at least one pathogen in a higher proportion of patients than conventional diagnostics (91.4% vs. 64.5%), with an overall good concordance with results of conventional cultures. However, when using MSP, the possibility of overdiagnosis due to the higher sensitivity and the lack of coverage of some pathogens (e.g. in this case fungi, herpesviruses and SARS-CoV-2) should also be considered in the clinical evaluation of the patient.

As expected, differences in pneumonia type explained the microbial spectrum: we observed a higher prevalence of MDR organisms (*K. pneumoniae* 28%, *P. aeruginosa* 24%, *A. baumannii* complex 11%), while Markussen’s cohort was dominated by community-associated pathogens such as *H. influenzae* and SARS-CoV-2.

In CAP, empiric regimens based on β-lactams and macrolides are generally recommended (REF). Conversely, in CAP-MDR, empirical therapy for CAP is often inappropriate, as confirmed by our findings and prior literature [11, 12]. This has key implications for antimicrobial stewardship: in our study, early MSP results enabled de-escalation in 21.5% of cases, reducing unnecessary anti-MRSA or anti-pseudomonal use, and escalation in 44.1%. Markussen et al., however, did not assess the impact of MSP results on therapy adjustments, limiting direct comparison with our findings.

Regarding the primary outcome—the rate of antibiotic therapy modification based on MSP testing results—our study found a modification rate of 65.6%. Notably, this finding is consistent with rates reported in the literature in other clinical settings, particularly in ICUs [18, 22, 32–36] (Table 4). However, as shown in Table 4, the proportions of antibiotic escalation and de-escalation vary widely among studies. This heterogeneity is probably attributable to differences in patient populations and case definitions, with HAP often being the dominant clinical entity investigated [22, 35].

**Table 4:** Literature Review: Rates of modification of antibiotic therapy

Author	Year	Setting	Pneumonia	Number of patients	Rate of change in therapy
Monard et al. [18]	2020	ICU	HAP, CAP, VAP	*N* = 159	77%
Gadsby et al. [22]	2015	ICU	HAP, VAP, CAP	*N* = 74	60.3%: escalation 40%, de-escalation 54.3%
Peiffer-Smadja et al. [29]	2020	ICU	HAP, VAP	*N* = 85	66%: escalation 21%, de-scalation 39%, optimization 3%
Pedro Garrido et al. [30]	2023	ICU	VAP	*N* = 93	71.4%: escalation 23.6%, de-escalation 76.4%
Buchan et al. [31]	2020	Multiple care settings	HAP, VAP	*N* = 259	70.7%: escalation 4.3%, de- escalation 48.2%
Miller et al. [32]	2023	ICU	HAP, CAP, VAP	*N* = 58	89.9%: escalation 10.3%, de-escalation 69.9%, discontinuation 10.3%.
Esplund et al. [33]	2022	ICU	-	154	54%: escalation 23%, de-escalation 77%.

In our cohort, MSP testing yielded a positivity rate of 91.4%, underscoring its ability to provide rapid pathogen identification in patients with moderate-to-severe pneumonia and risk factors for MDR infection. This rate is comparable, or even higher, to those reported in other European case series [17, 37–39]. A French multicentre study by Gastli et al. [40] reported a lower positivity rate (74.6%). Several factors may explain this discrepancy. First, patient populations differed: our cohort included many with moderate-to-severe pneumonia and significant healthcare exposure, conditions linked to MDR pathogens, whereas Gastli et al. examined a more heterogeneous group, including community-acquired infections of variable severity. Second, sample timing was critical. We obtained respiratory specimens promptly at ED presentation, likely increasing sensitivity, while Gastli et al. did not report timing; delays and prior antibiotics may have reduced yield. Finally, sample type and quality varied: nearly all of our specimens were high-quality bronchoscopic samples, enhancing accuracy compared with less targeted methods.

An interesting comparison arises with data from ICU patients, where MSP testing detected polymicrobial aetiologies in 62% of cases [40], similar to the 59.1% observed in our cohort. This similarity supports the notion that CAP-MDR share epidemiological and microbiological features with MDR-related entities such as HAP and VAP. Whether this high prevalence of polymicrobial findings reflects true infection or colonisation remains uncertain. In particular, the clinical significance of low-copy pathogen counts (10^4–10^5 Bin (cp/ml)) detected by MSP is unclear and warrants further investigation.

Regarding the prevalence of resistance genes, it is also important to consider the findings of Maataoui et al. [41], who applied MSP testing to manage respiratory infections in COVID-19 ICU patients and documented resistance gene rates exceeding 60% in HAP and VAP. Kreitmann and Nseir [42] reported similar results when evaluating multiplex PCR in ICU-acquired VAP. The higher MDR prevalence reported in those studies, compared with the 43.8% observed in our cohort, is consistent with the greater MDR burden typically seen in HAP and VAP, especially in intensive-care settings.

Our findings may have important clinical implications for the management CAP-MDR. In our experience, early use of MSP testing in ED showed a high diagnostic yield that led to rapid modifications in antibiotic therapy in a substantial number of patients (65.6%). Moreover, the adoption of MSP not only enables rapid and accurate identification of pneumonia pathogens, but also of key resistance genes, and this approach allowed for reduction of broad-spectrum antibiotics in a significant number of patients (21.5%). Moreover, the rapid turnaround time for microbiological diagnosis enables targeted treatment to be instituted directly in the ED rather than days after hospitalisation, as is typical with conventional culture-based diagnostics.

### Limitations

The main limitation of this study is its single-centre, observational design, conducted in a highly specialized, university-affiliated teaching hospital. This restricts the generalizability of the findings, particularly to EDs with fewer resources or a less complex case mix. In fact, the exclusive inclusion of patients who underwent MSP testing precludes direct comparison with those who did not receive early MSP testing, limiting the ability to assess impacts on clinical outcomes such as hospital length of stay, readmission rates, and mortality. These outcomes should be evaluated in adequately powered, prospective trials. Moreover, since enrolment focused on patients with moderate to severe CAP-MDR requiring advanced therapies, generalizability to milder cases is limited.

## Conclusions

In conclusion, we found that early testing with MSP on lower respiratory tract samples from patients with moderate to severe CAP-MDR directly in the ED is feasible and safe. It enabled rapid optimization of empiric antimicrobial therapy in more than 60% of patients. Further studies with larger cohorts and a broader range of participating centres are needed to assess clinical impact and cost-effectiveness.

## Supplementary Information

Below is the link to the electronic supplementary material.

Supplementary Material 1 (DOC 206 KB)

## Data Availability

The data that support the findings of this study are not publicly available due to their containing information that could compromise the privacy of research participants but could available from LP upon reasonable request.

## Declarations

### Study approval statement

The study protocol (CEAVC 26198_oss) was approved by the Tuscany Regional Ethics Committee (ClinicalTrials.gov: NCT06506617).
