# Supplementary material for: Rapid testing with molecular syndromic panels of patients presenting at the emergency department with pneumonia at risk for multidrug-resistant pathogens
Source: Eur J Clin Microbiol Infect Dis. 2025 Dec 10;45(3):863–75. doi: 10.1007/s10096-025-05376-2 (PMC12988998; doi:10.1007/s10096-025-05376-2)
Supplement: Supplementary file 1 — Supplementary Material 1 (DOC 206 KB) [file 10096_2025_5376_MOESM1_ESM.doc]

**FilmArray® Pneumonia Plus Panel (bioMérieux)**

The FilmArray® Pneumonia Plus Panel (bioMérieux) uses a self-contained, single-use pouch that incorporates all reagents required for nucleic-acid extraction, amplification, and detection. The system is designed to process BAL-like and sputum-like specimens without the need for pre-treatment or manual nucleic-acid purification. Once the sample is mixed with the proprietary buffer and injected into the pouch, the device performs all analytical steps automatically.

Inside the pouch, the specimen first undergoes mechanical lysis through bead-beating, followed by magnetic-bead–based extraction and purification of nucleic acids. The analytical workflow relies on a nested, multiplex PCR strategy: an initial highly multiplexed amplification step (PCR1) generates a broad pool of amplicons, which is subsequently distributed into multiple singleplex second-stage PCR reactions (PCR2), each specific for a single target. Detection is based on post-PCR melting-curve analysis, allowing the software to identify each pathogen or resistance gene according to its characteristic melting temperature.

For bacterial targets included in the semi-quantitative section of the panel, the system also integrates real-time amplification kinetics using an internal quantified standard. This enables the platform to provide an estimated genomic copy number per millilitre, reported in predefined bins, which helps approximate pathogen abundance. The full analytical process is completed in about one hour.

The pathogens and resistance genes are reported in table S1.

Table S1

| **Bacteria (Semi-Quantitative)** | **Atypical Bacteria (Qualitative)** | **Viruses** | **Antimicrobial Resistance Genes** |
| --- | --- | --- | --- |
| *Acinetobacter calcoaceticus-baumannii complex*  *Enterobacter cloacae*  *Escherichia coli*  *Haemophilus influenzae*  *Klebsiella aerogenes*  *Klebsiella oxytoca*  *Klebsiella pneumoniae group*  *Moraxella catarrhalis*  *Proteus spp.*  *Pseudomonas aeruginosa*  *Serratia marcescens*  *Staphylococcus aureus*  *Streptococcus agalactiae*  *Streptococcus pneumoniae*  *Streptococcus pyogenes* | *Legionella pneumophila*  *Mycoplasma pneumoniae*  *Chlamydia pneumoniae* | *Influenza A*  *Influenza B*  *Adenovirus*  *Coronavirus (non SARS-coV)*  *Parainfluenza virus*  *Respiratory Syncytial Virus*  *Human Rhinovirus/Enterovirus*  *Human Metapneumovirus*  *MERS-CoV* | *CTX-M*  *KPC*  *NDM*  *Oxa48-like*  *VIM*  *IMP*  *mecA/C and MREJ* |

**Reference standard**

Lower respiratory tract specimens were processed using conventional culture methods for the detection of bacterial and fungal pathogens. For this purpose, ten microliters of respiratory samples were inoculated onto non-selective media, including Columbia blood agar (CBA), chocolate agar (CHA) and CHROMagar^TM (^Candida (CAC), and incubated at 37°C in aerobic atmosphere (CBA and CAC) or in a 5% CO₂ atmosphere (CHA). Median were from Biomerieux and ThermoFisher. Bacterial growth was assessed after 24 and 48 hours of incubation. Bacterial colonies were identified by matrix-assisted laser desorption ionization–time of flight mass spectrometry MALDI-ToF (MALDI Biotyper®, Bruker, US), and potential pathogens were reported semi-quantitatively as colony-forming units per milliliter (CFU/mL). Antibiotic susceptibility testing (AST) was performed using semiautomated systems based on broth microdilution, including BD Phoenix (Becton-Dickinson, US) and (Merlin Diagnostika GmbH, Germany).

Urinary antigen testing for *Streptococcus pneumoniae* and *Legionella pneumophila* were performed by fluorescence immunoassay in urine samples (STANDARD F *S.pneumoniae* and *Legionella* Ag FIA, SD BIOSENSOR). *Aspergillus* galactomannan assay on BAL was performed by chemiluminescent immunoassay (Monotest Virclia- Vircell SL). Nucleic acid amplification tests for the detection of viral pathogens (*SARS-CoV-2,*  *Herpesviruses*) and fungal pathogens (*Aspergillus spp.* and *Pneumocystis jiroveci)* not included in the MSP, were performed by RT-PCR systems (Elite MGB® Kit Elite Ingenius – ElithechGroup, and Allplex™SARS-CoV-2Seegene) according to manufacturer’s instructions. *SARS-CoV-2* detection in nasopharyngeal swabs was performed either by chemiluminescent enzyme immunoassay for viral antigen (CLEIA- Lumipulse® SARS-CoV-Ag- Fujirebio) or by immunochromatographic point of care tests directly in the ED (Alifax srl, Fiatest).

***
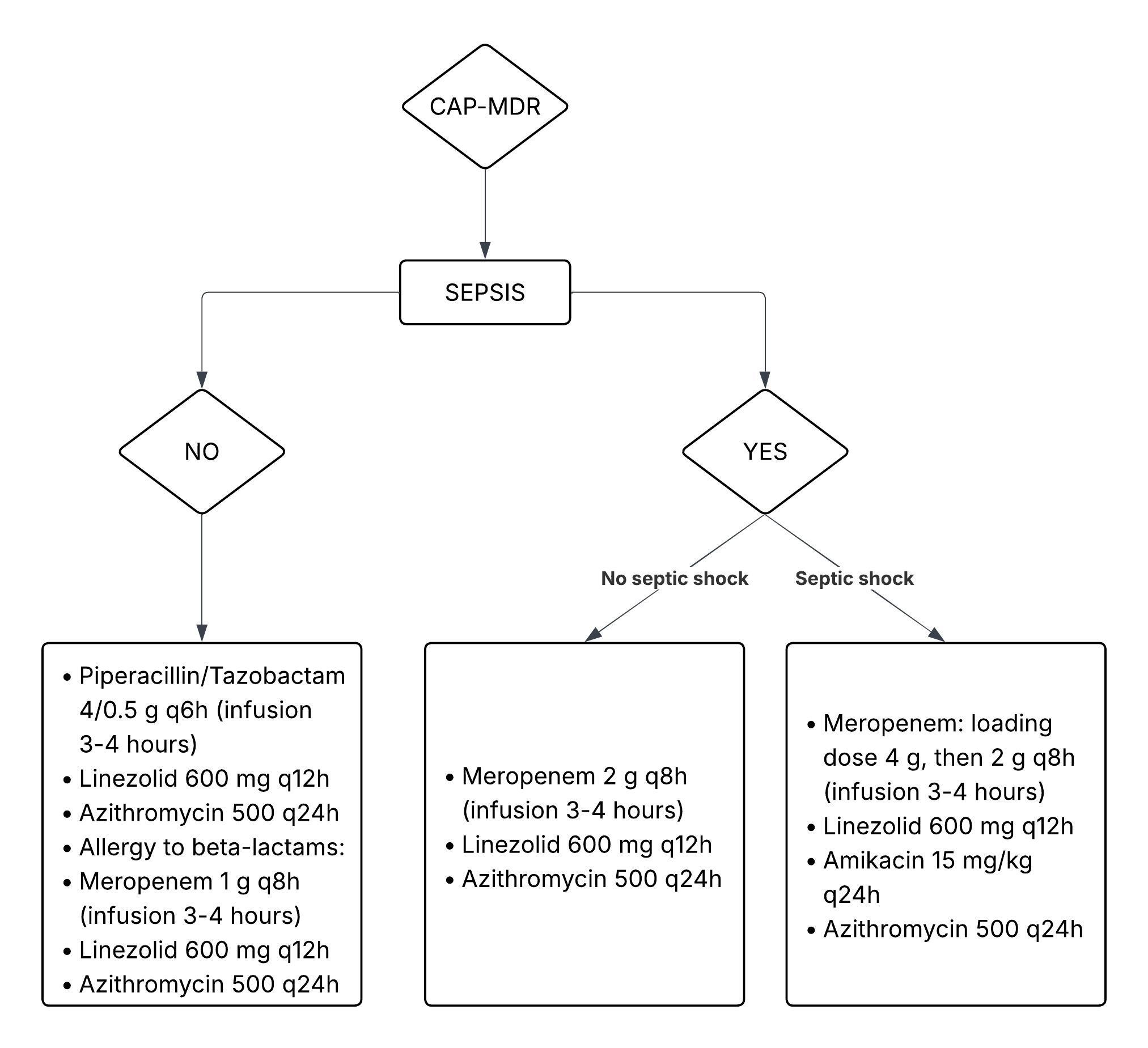
***

**eFigure 1e** – R**ecommendations for empiric antibiotic therapy in patients with CAP-MDR, according to institutional protocols of Careggi University Hospital.** As an alternative to linezolid-based antibiotic therapy, vancomycin may be used at a dose of 15 mg/kg every 12 hours (maximum 2 g/day), to be administered over 3–4 hours following appropriate dilution in accordance with the drug’s summary of product characteristics. Septic shock is defined as the presence of sepsis requiring vasopressor support to maintain a mean arterial pressure ≥65 mmHg despite adequate fluid resuscitation, and with serum lactate levels <2 mmol/L.

| **Additional microbiological tests** | | | |
| --- | --- | --- | --- |
|  | N° tests/total | Positive tests:  N (%) | Positive blood cultures and MSP for same pathogen |
| **Blood cultures** | 58/93 | 12/93 (13%) | 4 |
| **Urinary antigen tests (*S. pneumoniae*)** | 71/93 | 9/93 (9.6%) | |
| **Urinary antigen tests (*Legionella p.)*** | 70/93 | 2/93 (2%) | |
| **Nasopharyngeal swabs for SARS-COV2** | 93/93 | 5/93 (5.3%) | |
| ***Aspergillus* *spp*. PCR (BAL)** | 30/93 | 9/93 (9.6%) | |
| ***Pneumocystis spp*. PCR (BAL)** | 27/93 | 9/93 (9.6%) | |
| **Galactomannan (BAL)** | 28/93 | 18/93 (19.3%) | |

Table S2 - Additional microbiological tests

| Pathogens isolated | N° (total 11) |
| --- | --- |
| *Escherichia Coli* | 2 |
| *Staphylococcus Aureus* | 2 |
| *Enterococcus faecalis* | 2 |
| *Candida albicans* | 2 |
| *Proteus mirabilis* | 1 |
| *Klebsiella pneumoniae* | 1 |
| *Acinetobacter baumannii* | 1 |

Table S3 – Blood culture isolated pathogens. In 2 cases blood cultures were positive for *Pseudomonas oryzihabitans* and *Bacillus megaterium + Corynebacterium aurimucosum* that were considered contaminants
